# Pseudoepitheliomatous Hyperplasia of the Medial Thigh with Dystrophic Calcification and Metaplastic Ossification: A Case Report

**DOI:** 10.7759/cureus.108631

**Published:** 2026-05-11

**Authors:** Linda M Oster, Michael C Zornes, Patricia Palanca, Adelso Tejada Jackson, Dmitriy Kim

**Affiliations:** 1 Department of Surgery, Ross University School of Medicine, Miramar, USA; 2 Department of Surgery, St. John’s Episcopal Hospital, Far Rockaway, USA

**Keywords:** cutaneous lesion, epidermal proliferation, non-neoplastic skin lesion, pseudoepitheliomatous hyperplasia, squamous cell carcinoma of the skin

## Abstract

Pseudoepitheliomatous hyperplasia (PEH) is a benign, reactive epidermal proliferation that may histologically mimic squamous cell carcinoma (SCC) due to its pseudo-infiltrative growth pattern. While typically associated with chronic inflammation, trauma, or infection, it is considered to be benign and does not warrant oncological management. Here, we present the case of a 71-year-old African American female with morbid obesity among other comorbidities who presented with a painful, ulcerated right medial thigh mass of 10 years’ duration. The patient, a nursing home resident, reported significant localized pain upon presentation. Imaging revealed a large soft-tissue mass with calcification. Biopsy revealed ulcerated skin and extensively fibrosed subcutaneous tissue exhibiting dystrophic calcification, ossification, and granulation tissue, without features of malignancy. Final pathology confirmed PEH. Following the biopsy and confirmation of the benign nature of the lesion, the patient’s management focused on conservative wound care and pain control, successfully avoiding the morbidity of radical surgical resection. This case underscores the diagnostic challenge in differentiating PEH from SCC and highlights the importance of making a clear diagnosis to provide great patient care and avoid unnecessary treatments that carry risk.

## Introduction

Pseudoepitheliomatous hyperplasia (PEH) is defined as a benign reactive proliferation of the epidermis characterized by the downward projection of squamous epithelium into the dermis. Histologically, its pseudo-infiltrative growth pattern can closely resemble squamous cell carcinoma (SCC) [1-4]. PEH may arise in association with chronic inflammation, infection, trauma, or neoplasia, and is even seen adjacent to or in conjunction with SCC [2-6]. Additionally, while PEH has been documented in many anatomical locations (most notably the oral mucosa, the vulva, and increasingly in cutaneous reactions to red ink tattoos), its presentation in the lower extremities is unusual and potentially under-recognized. More specifically, the clinical and pathological similarities that exist between PEH and SCC make thorough evaluation paramount to avoid misdiagnosis and potential overtreatment [3,4,7-11]. To demonstrate the novelty of this case and provide a comparison with previously reported instances of PEH in the literature, we have summarized a brief literature search performed using PubMed. In total, 45 case reports published between 2006 and 2026 were included in the review, excluding all non-human case reports and articles written in a language other than English.

## Case presentation

A 71-year-old African American female with a past medical history of morbid obesity, hyperlipidemia, hypothyroidism, hypertension, uterine cancer s/p hysterectomy, and depression presented to the emergency department at a community hospital in Far Rockaway, New York, in October 2024. The patient presented to the hospital for a mass on her right medial thigh, which had been present for 10 years and had recently become painful and ulcerated over a three-week period despite routine wound care. The patient reported that the mass had not grown in size during this time. On assessment, the patient was hemodynamically stable, afebrile, and alert. On initial examination, the area of ulceration of the right medial thigh measured 4 × 4 cm, was non-foul-smelling, had no discharge, and was tender to palpation.

**Figure 1:**
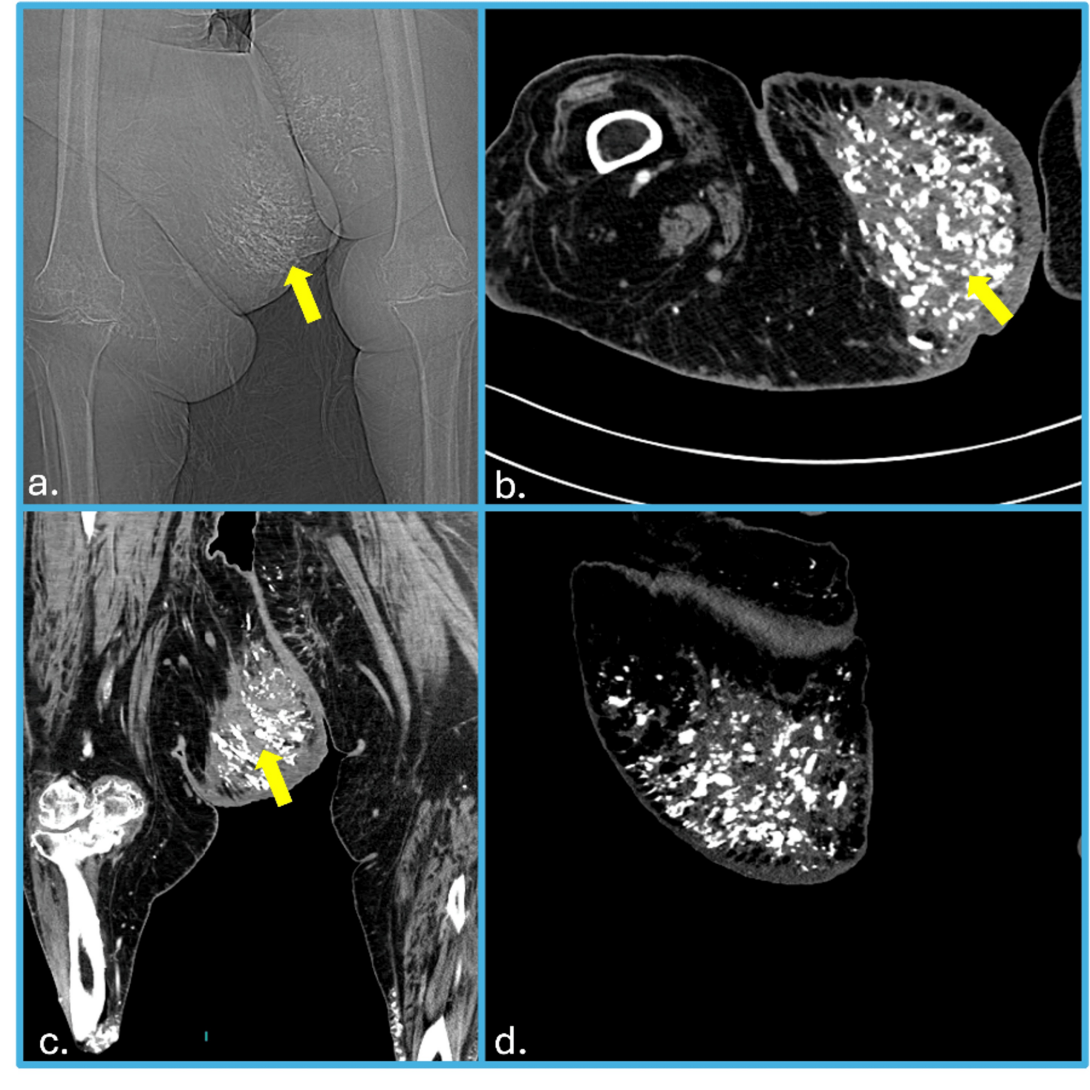
CT imaging of the right lower extremity. (a) Topogram of the lesion with the yellow arrow pointing to the mass grossly seen on the right medial thigh. (b) Axial soft-tissue view of the right lower extremity with the yellow arrow pointing to the mass. (c) Coronal soft-tissue view of the right lower extremity with the yellow arrow pointing to the mass. (d) A sagittal soft-tissue view of the right lower extremity mass.

Laboratory findings on admission were significant for hyperlipidemia (Table 1). Notably, metabolic markers, including calcium (9.3 mg/dL) and phosphate (3.6 mg/dL), were normal. CT imaging (Figure 1) showed a large soft-tissue mass with coarse, calcific deposits overlying skin thickening and ulceration associated with a mass in the right proximal thigh measuring 17 × 8.8 cm. The CT topogram (Figure 1a) provided a definitive gross view of the mass’s location and scale, while the axial and coronal views (Figures 1b, 1c) delineated the extensive internal mineralization.

**Table 1:** Laboratory findings. WBC = white blood cell; LDL = low-density lipoprotein; VLDL = very-low-density lipoprotein; HDL = high-density lipoprotein; WNL = within normal limit

Category	Test	Result	Reference range	Interpretation
Inflammation and infection	WBC count	4.9 × 10³/L	5.2–12.4 × 10³/L	Low
Metabolic Panel	Albumin	3.3 g/dL	3.5–5.0 g/dL	Low
	Calcium	9.3 mg/dL	8.4–10.2 mg/dL	WNL
	Phosphate	3.6 mg/dL	2.5–4.5 mg/dL	WNL
	Sodium	139 mmol/L	137-145 mmol/L	WNL
	Potassium	4.2 mmol/L	3.5–5.1 mmol/L	WNL
	Chloride	111 mmol/L	98–107 mmol/L	High
Iron studies	Hemoglobin	13.4 g/dL	12.0–16.0 g/dL (F)	Low
	Hematocrit	35.5	37–37	Low
Lipids	Total cholesterol	211 mg/dL	<200 mg/dL	Borderline High
	LDL cholesterol	150 mg/dL	<100 mg/dL	High
	VLDL cholesterol	21.4 mg/dL	5–40 mg/dL	WNL
	HDL (direct)	30 mg/dL	≥60 mg/dL	Low
	Cholesterol/HDL ratio	7	<4	Elevated cardiovascular risk

Intraoperatively, a full-thickness biopsy was obtained. Specimens were fixed in formalin and appeared as an aggregate of soft-to-hard tissue fragments measuring 2.0 × 1.5 × 0.5 cm. Hematoxylin and eosin staining was performed.

Ultimately, due to the high burden of comorbidities and benign nature of the lesion, the patient underwent conservative management with both inpatient wound care and pain management and was referred for regular outpatient wound care follow-up.

Pathology findings

**Figure 2:**
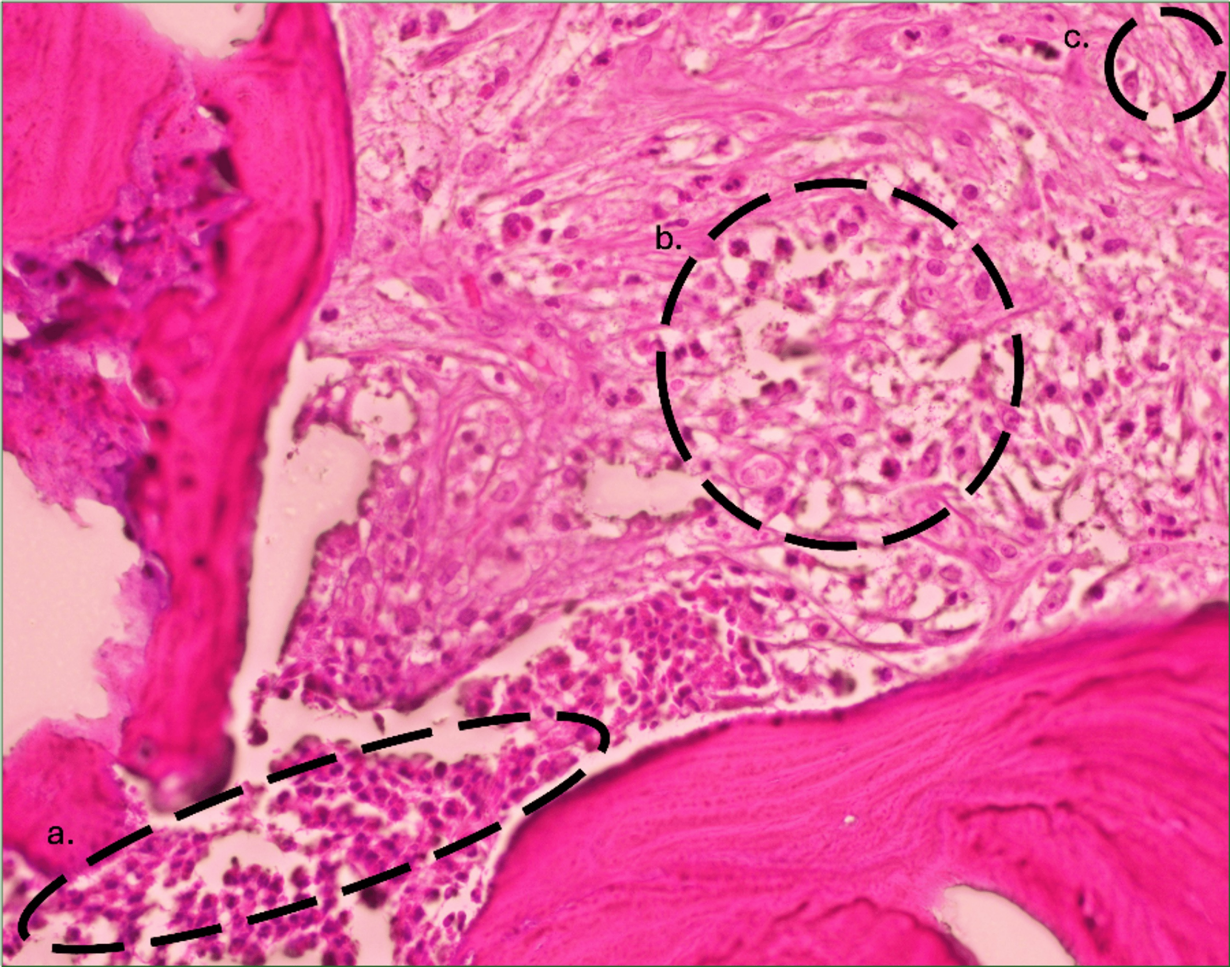
Histologic section of ulcerated subcutaneous tissue demonstrating some key reactive changes seen in pseudoepitheliomatous hyperplasia. Hematoxylin and eosin stain, original magnification ×200. (a) Mature lamellar bone formation with surrounding inflammatory infiltrate (circled), consistent with reactive bone formation. (b) Central area of granulation tissue, characterized by a dense mixed inflammatory infiltrate, including neutrophil, lymphocytes, and vascular proliferation. (c) Upper right portion shows dense fibrosis, with eosinophilic collagen bundles indicating a chronic reparative response.

On examination, pathological analysis revealed ulcerated skin along with extensively fibrosed subcutaneous tissue, calcification, and ossification with acute and chronic inflammation and granulation tissue (Figure 2). The aforementioned findings were largely non-neoplastic in nature. Final pathology revealed PEH, ulceration, fibrinopurulent exudate, granulation tissue reaction, fat necrosis, and dystrophic calcification.

## Discussion

PEH is a reactive epithelial proliferation often associated with chronic inflammation, infection, trauma, or cancer [1-4]. In this patient, the likely precipitating factor for the development of PEH was a 10-year course of mechanical friction due to high adiposity in the lower extremities secondary to morbid obesity and limited mobility. For a patient who presented with such a long-standing lesion, differentiation between PEH and SCC was vital to provide appropriate care.

Interestingly, the occurrence of PEH in the lower extremity, particularly in association with the combination of ulceration and dystrophic calcification, is rare and may be underreported (Table 2). Calcification itself is often seen in the setting of chronic wounds or inflammation [6,7], and in this case may also be a manifestation of prolonged tissue damage. Furthermore, histologic analysis of the specimen not only demonstrated calcification but also revealed the presence of ossification. The possibility of metaplastic ossification as a downstream consequence of chronic inflammation not only complicates interpretation for the clinical team but is even more rarely documented in the context of PEH [4,8].

**Table 2:** Comparative analysis of reported pseudoepitheliomatous hyperplasia (PEH) cases. A brief summary of PEH cases published on PubMed between 2006 and 2026, excluding reports not published in the English language and those reporting on PEH in animals.

	Study	Location	Precipitating factor/trigger	Primary histopathological findings
Head and neck	Zhang et al. [12]	Auricle	Chronic inflammation (unspecified)	Reactive epidermal hyperplasia requiring complex surgical reconstruction; mimics invasive squamous cell carcinoma (SCC)
	Nayak et al.; Thorat et al.; Xiang et al. [3,13,14]	Gingival/Oral	Periodontal disease; lung neoplasia; or general review	Thorat et al. (2011) noted primary gingival PEH with periodontal findings; Xiang et al. (2012) described a “giant” presentation associated with lung SCC; Nayak et al. (2015) reported a clinical review of diagnostic criteria in oral lesions
	Raposo et al. [15]	Larynx	Chronic inflammation (unspecified)	Extreme reactive hyperplasia requiring aggressive intervention, including total laryngectomy
	Val-Bernal et al. [16]	Lingual	Subgemmal neurogenous plaques	Incidental pseudomalignant epidermal growth and reactive changes associated with neurogenous plaques
	Everett et al. [17]	Mandible	Prior oncologic therapy (post-SCC treatment)	Reactive epidermal hyperplasia arising in the mandible following prior treatment for malignancy
	Nonomura et al. [18]	Nasal	Extranodal NK/T-cell lymphoma	Reactive epidermal hyperplasia presenting a diagnostic pitfall for SCC adjacent to lymphoma
Ocular	Joshi et al. [19]	Ocular/Orbital: canalicular	Chronic inflammation	Novel description of isolated canalicular reactive epidermal hyperplasia in the ocular adnexa
	Fatima et al. [20]; Mohebbi et al. [21]	Ocular/Orbital: ocular surface/limbal	Cultivated limbal epithelium transplantation; episcleritis	Reactive hyperplasia mimicking ocular surface squamous neoplasia (OSSN) following surgical transplantation or inflammatory episcleritis
	Doctor et al. [22]	Ocular/Orbital: orbital	Probable IgG4-related orbital disease	Exuberant ocular surface reactive changes masking underlying systemic/orbital inflammatory disease
	Bothra and Ali [23]	Ocular/Orbital: punctal	Chronic inflammation	Reactive epidermal hyperplasia mimicking a mass lesion of the lacrimal punctum
Cutaneous	Amsbaugh et al.; Fischer et al.); Kamalapirat et al.; Quintella et al. [24-27]	Skin: infectious/inflammatory	Chronic infection (leprosy, sporotrichosis, leishmaniasis, bacillary angiomatosis)	Pathogen-induced epidermal hyperplasia; features include transepidermal elimination (leprosy) or lymphocutaneous spread (sporotrichosis)
	Meani et al.; Parsons & Ryder; Pusiol et al.; Speziali et al. [28-31]	Skin: inflammatory conditions	Lichen planus; allergic contact dermatitis; HIV/AIDS	Reactive epidermal proliferation secondary to chronic inflammatory dermatoses or immunocompromised states
	Ansari et al.; Biswas et al.; Ling et al.; Rossi et al.; Shafi et al.; Vucić et al.; Wang et al. [32-38]	Skin: neoplasia and lymphoma	Malignancy (melanoma, cutaneous lymphoma, mycosis fungoides, trichoepithelioma)	Reactive proliferation adjacent to or overlying neoplastic lesions; a frequent diagnostic pitfall for invasive SCC due to pseudo-infiltrative growth
	Badavanis et al.; Breza et al.; Conejero et al.; Conti et al.; Cui et al.; de Roeck et al.; Kiss et al.; Tammaro et al. [39-46]	Skin: red ink tattoo	Red ink/tattoo pigment	Exuberant reactive changes and late-onset epidermal hyperplasia secondary to exogenous tattoo pigment
	Blum & D’Souza [8]	Skin: trauma/foreign body	Displaced metallic orthopedic implant	Reactive epidermal hyperplasia secondary to chronic irritation and trauma from metallic foreign bodies
Other	Desimone et al. [47]	Breast	Granular cell tumor	Exuberant reactive epidermal proliferation adjacent to benign neoplasia of the breast
	Han et al. [48]	Esophagus	Lye-induced chemical trauma	Reactive changes mimicking esophageal SCC secondary to chronic stricture and chemical injury
	Eandi et al. [49]	Urinary bladder	Granular cell tumor	Visceral reactive hyperplasia co-localizing with adenocarcinoma or benign neoplasia
	Frimer et al.; Lee et al.; Tangjitgamol et al. [4,50,51]	Vulvar/Genital	HIV/AIDS; lichen sclerosus; Herpes simplex virus-II	Reactive changes in the genital tract mimicking vulvar cancer; associated with chronic dermatoses or viral infections in immunocompromised patients

Histologically, PEH is characterized by irregular acanthosis, elongation of rete ridges, and a pseudo-infiltrative growth pattern that may mimic SCC. However, unlike SCC, PEH lacks cytologic atypia, abnormal mitoses, and actual invasion of surrounding tissues [3]. As illustrated by Chakrabarti et al. [5], these nuanced but crucial differences often require multiple, deep biopsies, tissue culture, immunohistochemistry, and/or immunofluorescence [3], and it is important that clinicians do not overlook the distinction between the two conditions. PEH is considered to be benign, and the standard of care does not include wide excision or lymph node evaluation unless a confirmed malignancy is present. However, despite its benign nature, overtreatment is a well-documented risk due to its close resemblance to SCC, and this makes multidisciplinary collaboration between dermatology, pathology, surgery, and radiology essential in ambiguous cases. Additionally, while surgical excision with clear margins is indicated to avoid recurrence, the clinician must balance this with a holistic approach for each patient. When considering surgical management for either oncologic management of SCC or management of recurrence in PEH, possible morbidity, particularly in elderly or comorbid patients, where radical surgeries may not be well-tolerated, must be considered.

In summary, this case highlights an unusual presentation of PEH resembling ulcerated SCC on physical examination. Uniquely, this case of PEH revealed dystrophic calcification and ossification on histological evaluation, which has not been documented in the recent body of published work. This case not only adds to a growing body of literature on this potentially painful and socially devastating condition but also emphasizes the importance of differentiating PEH from malignancies such as SCC to deliver appropriate and timely care to patients.

## Conclusions

PEH remains a diagnostic challenge due to its histologic resemblance to SCC, particularly in chronic, ulcerated lesions with reactive tissue changes. This case highlights an uncommon presentation of PEH involving the thigh with significant dystrophic calcification and ossification, features that are rarely documented together. Recognition of these benign reactive processes is critical to prevent unnecessary aggressive surgical intervention or oncologic management. Clinicians should maintain a high index of suspicion and pursue thorough histopathologic correlation when evaluating chronic cutaneous lesions with atypical features. Ultimately, multidisciplinary collaboration between dermatology, pathology, radiology, and surgery ensures accurate diagnosis and optimal patient outcomes in such diagnostically complex cases.
